# Geospatial Disparities and the Underlying Causes of Major Cancers for Women in Taiwan

**DOI:** 10.3390/ijerph110605613

**Published:** 2014-05-26

**Authors:** Chi-Ting Chiang, Ie-Bin Lian, Ying-Fang Chang, Tsun-Kuo Chang

**Affiliations:** 1 Green Energy and Environment Research Laboratories, Industrial Technology Research Institute, No. 195, Section 4, Chung Hsing Road, Chutung, Hsinchu 310, Taiwan; E-Mail: changyingfang@itri.org.tw; 2 Graduate Institute of Statistics and Information Science, National Changhua University of Education, No. 1, Jin-De Road, Changhua 500, Taiwan; E-Mail: maiblian@cc.ncue.edu.tw; 3 Department of Bioenvironmental Systems Engineering, National Taiwan University, No. 1, Section 4, Roosevelt Road, Taipei City 106, Taiwan; E-Mail: tknchang@ntu.edu.tw

**Keywords:** spatial autocorrelation, geographic information system, principal component analysis, cancer, environmental pollution, Taiwan

## Abstract

Some specific types of cancer still pose a severe threat to the health of Taiwanese women. This study focuses on determining the geographical locations of hot spots and causal factors related to the major categories of cancers in Taiwanese women. Cancer mortality data from 1972 to 2001 of 346 townships in Taiwan were obtained from the Atlas of Cancer Mortality. Principal component analysis was conducted to determine the primary categories of female cancers. The spatial patterns of hot spots and cold spots for each major cancer category were identified using the local indicator of spatial association. Finally, the regional differences between the hot spots and cold spots were compared to confirm the possible factors causing cancer throughout Taiwan. A total of 21 cancer types in women were divided into seven major categories, which accounted for 68.0% of the total variance. The results from the spatial autocorrelation analysis showed significant spatial clusters of the cancer categories. Based on the overall consistency of results between this study and those of previous research, this study further identified the high-risk locations and some specific risk factors for major cancer types among Taiwanese women.

## 1. Introduction

**Figure 1:**
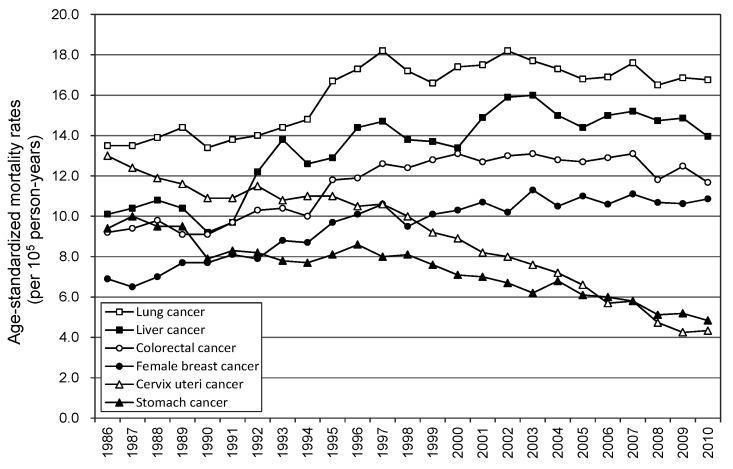
Secular trends in age-standardized mortality rates of lung, liver, colorectal, breast, and cervix uteri cancers for females in Taiwan, 1986–2009.

Since the early 1980s, cancer has ranked first among the 10 leading causes of death in Taiwan, and the average age at death from cancer has shown a persistent downward trend on a yearly basis in recent years. Cancer was responsible for 28.1% of 142,240 deaths recorded in Taiwan in 2009, and one death occurs from cancer every 14 minutes [1]. In Taiwan, liver, lung, and colorectal cancers are the most common types of cancer for both sexes. According to annual cancer reports from the Taiwan Ministry of Health and Welfare (MOHW), lung cancer has surpassed breast cancer to become the most common cause of cancer among women from 1986 until the present. Between 2000 and 2009, the 9-year average mortality rate of lung cancer in women in Taiwan was 17.3 cases per 100,000 person-years, and the number of deaths caused by lung cancer increased from 1956 cases to 2615 cases per year, representing an increase of 33.7% [1]. Additionally, breast cancer is the most frequently diagnosed female-specific type of cancer in Taiwan, followed by cervix uteri cancer. In recent years, liver, lung, breast, and cervix uteri cancers accounted for nearly half (49%) of all cancer deaths in women in Taiwan [1]. Figure 1 shows upward trends in age-standardized mortality rates of female lung, liver, colorectal, and breast cancers in Taiwan from 1986 to 2010; by contrast, a downward trend in the mortality rate of cervix uteri cancer has been reported. Therefore, specific types of cancer still pose severe threats and challenges to women’s health in Taiwan.

The development of various types of cancer is complex and is attributed to multiple underlying risk factors including hereditary, sociodemographics, dietary, lifestyle, and environmental factors. However, certain carcinogenic substances have been linked to specific types of human cancer. Among these factors, the effect of tobacco smoking on cancer risk remains the most crucial discovery in the history of cancer epidemiology [2]. Numerous studies have indicated that tobacco use, particularly cigarette smoking (CS), is a major or predominating causative factor of tobacco-related cancers of the respiratory tract, oral cavity, and stomach [3,4]. Cancer likely results from several interacting factors. Although each type of cancer has its own set of unique causes and risk factors, some risk factors might cause the development of certain types of cancer, and are thus interrelated.

Spatial epidemiology focuses on describing and determining geographic variations in the relationship between diseases and risk factors, which has a long history dating back to the spot map of cholera cases in the London epidemic of 1854 developed by Snow [5]. Current geographic information systems (GIS) and spatial statistical methods allow epidemiologists to ascertain and address the spatial patterns of disease occurrence and transmission more effectively and completely. Numerous epidemiological studies have extensively used these spatial-analytical techniques to identify possible causes for the occurrence of specific diseases or epidemics in recent years. Clarifying the distribution of disease either in temporal, spatial, or spatial-temporal clusters is a foundation of epidemiology, which provides valuable clues for further in-depth studies into controlling and preventing of disease. Certain studies detecting cancer clusters at local, regional, and state levels have been performed to assess geographic variations in specific cancer incidence or mortality [6,7,8]. The purpose of epidemiology is to search for the causes of disease and, by inference, develop approaches for deterring or preventing disease [9]. However, a cancer cluster with an abnormally high incidence of a particular cancer is likely to be associated with particular factors. Certain studies have reported that clustering regions of high incidence or mortality rates of several types of cancer exhibit severe environmental pollution, demographic characteristics, or lower socioeconomic status [6,8,10,11,12,13].

We therefore categorized numerous types of cancer to identify and characterize geographical clusters of these major cancer categories with statistically significant high (hot spots) and low (cold spots) mortality rates for women at the township level in Taiwan. We compared the risk factors related to cancers, including the prevalence of smoking and betel quid chewing (BQC), environmental pollution status (based on heavy metals content in soil), degree of urbanization (based on the population density), and racial differences (based on the proportion of aboriginal population) to determine the regional differences between hot spots and cold spots for each cancer category.

## 2. Materials and Methods

### 2.1. Mortality Rates of Various Cancers Data

Township-specific information on the mortality rates for common types of female cancer were derived from the Atlas of Cancer Mortality in Taiwan constructed in 2003, which contains age-standardized mortality rates (ASMR) throughout Taiwan for the 1972−2001 period. The age-standardized incidence rate is defined as the incidence or mortality rate standardized according to age distribution based on year 2000 world standard population by World Health Organization (WHO). The 18 most common malignant cancer types among Taiwanese adults comprise lung and liver cancer; colon, rectum, rectosigmoid junction and anus (CRRJA) cancer; and oral cavity, stomach, esophagus, pancreas, colon, rectum, gallbladder, bladder, brain, kidney, larynx, bone, nasopharynx, intestine, and skin (non-melanoma) cancer. Female breast, ovarian, and cervical cancers were also introduced into this study, forming a total of 21 female-related cancers.

### 2.2. Age- and Sex-Specific Population

Certain cancers occur more frequently with advancing age. In Taiwan, most cases of colorectal cancer and esophagus cancer occur primarily between the ages of 50 and 70 years. Both breast and ovarian cancer are most often observed in women aged 50–70 years, and female cervical cancer peaks at 35–45 years of age. The age- and sex-specific population for each township in 2001 was obtained from the Department of Household Registration Affairs.

### 2.3. Taiwanese Aboriginal Population Data

A principal source of demographic data for the aboriginal population in this study was the census. Several culturally and ethno-linguistically distinct aboriginal tribes in Taiwan live primarily in the eastern plains and central mountains, totaling approximately 500,000 people. Data on the aboriginal population of each township across Taiwan in 2001 were retrieved from the Department of Household Registration Affairs.

### 2.4. Prevalence Rates of BQC and CS

The prevalence rates of BQC and CS for 2002 were estimated from the National Health Interview Survey (NHIS) data, which was performed cooperatively by the National Health Research Institutes (NHRI) and the Taiwan MOHW. It surveyed the prevalence rates of CS and BQC in randomly selected subjects, including 13,086 men and 12,473 women. The survey participants included 1,086 males and 1,473 females. Data on the prevalence rates of BQC and CS were studied at the township level.

### 2.5. Soil Heavy Metal Content

Soil data in this study were derived from a progressive, nationwide survey that determined the concentration in agricultural topsoil (0–15 cm) of arsenic (As), cadmium (Cd), chromium (Cr), copper (Cu), mercury (Hg), nickel (Ni), lead (Pb) and zinc (Zn), conducted by the Environmental Protection Administration (EPA) in Taiwan from 1986 to 1990. The total concentration of extractable As and Hg in the soil was determined using the aqua regia method, and that of the other six heavy metals was determined using the 0.1 N HCl extraction method. A grid cell size of 1,600 ha was used as the sampling unit and 936 soil samples were collected across Taiwan. The area-weighted mean value represented the soil heavy metal content in each township.

### 2.6. Principal Component Analysis

Principal component analysis (PCA) is appropriate when collecting a large set of potentially correlated variables for transforming them into a new and smaller subset of uncorrelated variables (called principal components, PCs) that account for most of the variance in raw data [14]. PCA is an optimal eigenvector-based linear dimension-reduction technique for multiclass data, in which eigenvalues quantify how much variance is explained by each PC, and an eigenvalue greater than one is often used as the criteria for determining which PCs are retained. The first extracted PC accounts for the maximum amount of total variance in the data, the second PC is the next largest amount, and so on. PC scores can be created for each observation in a data set based on the eigenvectors for each PC. PC loadings are correlation coefficients between the original variables and the PC scores, ranging from −1 to 1. The results of a PCA can decrease or eliminate multicollinearity among regressor variables when conducting regression analyses to facilitate follow-up analysis.

### 2.7. Spatial Autocorrelation Analysis

Spatial autocorrelation analysis is a relatively novel technique used to detect and quantify the patterns of disease distribution that can provide insights into disease epidemiology. Spatial autocorrelation exists when a value observed in one geographic location relies on the values of neighboring locations. In this study, 2 scales were used to measure the level of spatial autocorrelation. The Moran index (Moran’s ***I***), a measure of global-spatial autocorrelation or overall clustering, was used to estimate the magnitude of association in a data set [15]. The global Moran’s ***I*** determines an overall spatial-clustering propensity in a study area, however, it is unaware of the exact position of a cluster occurrence. The local Moran index (Moran’s ***I_i_***), a measure of spatial autocorrelation on a local scale that determines the potential contribution for each location to the global Moran’s ***I***, is called a local indicator of spatial association (LISA) developed by Anselin [16]. The LISA statistics are used to identify spatial outliers, including local homogeneous (hot and cold spots) or heterogeneous regions. The null hypothesis (H_0_) for testing spatial autocorrelation is that a variable value for a specific region is unassociated with neighboring regions, whereas the alternative hypothesis (H_a_) is that the neighboring regions have similar variable values. When the null hypothesis is rejected, the result is statistically significant and there is spatial autocorrelation.

We first conducted PCA to determine the major, crucial female-cancer categories in Taiwan. We subsequently identified and determined the spatial cluster patterns for each major cancer category by using the LISA method. Finally, we examined possible relevant factors (such as population density, proportion of aboriginal population, and the heavy metals content in soil) that might cause regional differences in cancer prevalence throughout Taiwan. The exploratory spatial data analyses were conducted using the GeoDa cluster-detection software program, Version 0.9.5-I, developed by Anselin. For statistical inference, the significance was tested using a Monte Carlo test with 999 permutations at a significance level of 0.05. A Student’s t-test was used for comparing regional differences between hot spots and cold spots for each cancer category in possible relevant factors.

## 3. Results and Discussion

### 3.1. Principal Component Analysis for Various Types of Cancer

We conducted PCA to determine the major PCs influencing the patterns of female cancer mortality in Taiwan. The PCs were extracted based on eigenvalues greater than 1, obtaining seven PCs. These seven PCs accounted for 68% of the total variance. Table 1 shows the results of PC loadings, eigenvalues, and the cumulative percentage of variation explained by each of these retained seven PCs after rotation.

**Table 1:** Results from the principal component analysis for female cancer types.

Cancer types	PC1	PC2	PC3	PC4	PC5	PC6	PC7
Bone	0.24	0.07	−0.14	0.14	−0.09	−0.12	***0.77***
Bladder	***0.95***	−0.03	0.06	0.00	0.04	−0.01	0.04
Brain	0.04	0.42	0.35	−0.22	0.32	−0.27	0.12
Colon	0.09	−0.12	***0.85***	0.02	−0.13	0.00	0.01
Rectum	0.01	−0.10	0.33	***0.67***	0.09	0.19	0.11
Esophagus	−0.08	***0.68***	−0.06	0.37	0.27	0.01	0.11
Intestine	0.05	0.10	0.00	***0.81***	0.05	−0.16	−0.05
Kidney	***0.92***	−0.01	0.11	0.02	−0.05	0.06	0.06
Liver	0.33	0.16	−0.16	−0.21	0.44	0.32	−0.01
Gallbladder	−0.06	−0.11	0.24	−0.09	−0.30	0.26	0.35
CRRJA ^a^	0.08	−0.15	***0.87***	0.34	−0.06	0.10	0.06
Larynx	0.07	***0.78***	−0.03	−0.12	−0.24	−0.07	−0.13
Nasopharynx	0.02	***0.71***	−0.12	−0.22	0.19	−0.05	−0.05
Oral cavity	0.01	***0.73***	−0.19	0.38	0.04	−0.07	−0.08
Pancreas	−0.09	−0.26	0.34	−0.14	0.12	0.21	0.54
Stomach	−0.03	0.44	−0.28	−0.01	0.58	0.12	0.20
Skin(non-melanoma)	***0.81***	−0.03	−0.12	0.09	−0.07	−0.14	0.02
Lung	***0.80***	0.11	0.13	−0.05	0.17	0.24	0.04
Breast	−0.03	−0.05	0.46	−0.07	0.17	***0.61***	−0.09
Cervical	0.00	−0.05	0.06	0.20	***0.77***	−0.07	−0.14
Ovarian	0.09	−0.10	−0.04	0.03	−0.08	***0.78***	0.08
Eigenvalue	3.56	3.37	2.17	1.68	1.36	1.14	1.00
% Total variance	16.95	16.04	10.31	8.01	6.46	5.43	4.78
Cumulative % variance	16.95	33.00	43.31	51.33	57.78	63.22	68.00 ^b^

Notes: (1) ^a^ CRRJA indicates colon, rectum, rectosigmoid junction and anus cancer. (2) ^b^ Total cumulative variance. (3) The PC loading whose absolute value is greater than 0.60 of the total variance were in bold italic.

PC loadings greater than 0.60 in absolute value were used to determine whether to include a variable (cancer type) in the PC. Based on this criterion, the first PC (PC1) explained 16.95% of the total variance, with strong positive loadings on bladder, kidney, skin (non-melanoma) and lung cancers. The second PC (PC2) comprised esophagus, larynx, nasopharynx, and oral cavity cancers, which explained 16.04% of the total variance. The third PC (PC3) comprised colon and CRRJA cancers, which explained 10.31% of the total variance. The fourth PC (PC4) comprised rectum and intestine cancers, which explained 8.01% of the total variance. The cumulative explanation value of the first through fourth PC exceeded 50%. Based on the PCA results, each PC was assigned a category name, shown in Table 2. The association of bladder, kidney, skin (non-melanoma) and lung cancers in PC1 reflects the maximal influence of the urinary system on cancer mortality in Taiwanese women. Esophagus, larynx, nasopharynx, and oral cavity cancers were dominated by PC2, which reflects the influence of head and neck cancers (HNC) on female cancer mortality. The category name is based on the characteristics of cancer types contained in each PC, such as Category 1, 2, and so on. Additionally, PC scores were the derived composite scores computed for each township based on the eigenvectors for each PC (category), which were used as the new variables in subsequent spatial analyses.

**Table 2:** Given the appropriate name of each cancer category.

Categories	Category name	Cancers types
1	Urinary system	Bladder, kidney, skin (non-melanoma), lung
2	Head & neck	Esophagus, larynx, nasopharynx, oral cavity
3	Colorectal *	Colon, CRRJA
4	Bowel	Rectum, intestine
5	Cervical	Cervical
6	Female sexual organs	Breast, ovarian
7	Bone	Bone

Note: * Colorectal cancer includes malignant tumors of the colon and/or the rectum.

### 3.2. Global Spatial Autocorrelation of Cancer Categories

We constructed a contiguity-based spatial weight for each township based on queen contiguity relationships, which defines spatial neighbors as areas with shared border and vertices [17]. Table 3 summarizes the results of the global Moran’s ***I*** analyses, indicating the statistically significant (*p* < 0.05) and positive global-spatial autocorrelation values for these 7 cancer categories. We performed further LISA analyses to identify the hot spots and cold spots for 7 major cancer categories.

**Table 3:** Global Moran’s ***I*** statistics of each principal component.

Categories	Category Name	Moran’s *I*	*p*-Value
1	Urinary system	0.401 *	0.001
2	Head & neck	0.519 *	0.001
3	Colorectal *	0.270 *	0.001
4	Bowel	0.095 *	0.004
5	Cervical	0.208 *	0.001
6	Female sexual rgans	0.269 *	0.001
7	Bone	0.063 *	0.026

Note: * *p* < 0.05.

### 3.3. Spatial Clustering for 7 Major Cancer Categories

**Figure 2:**
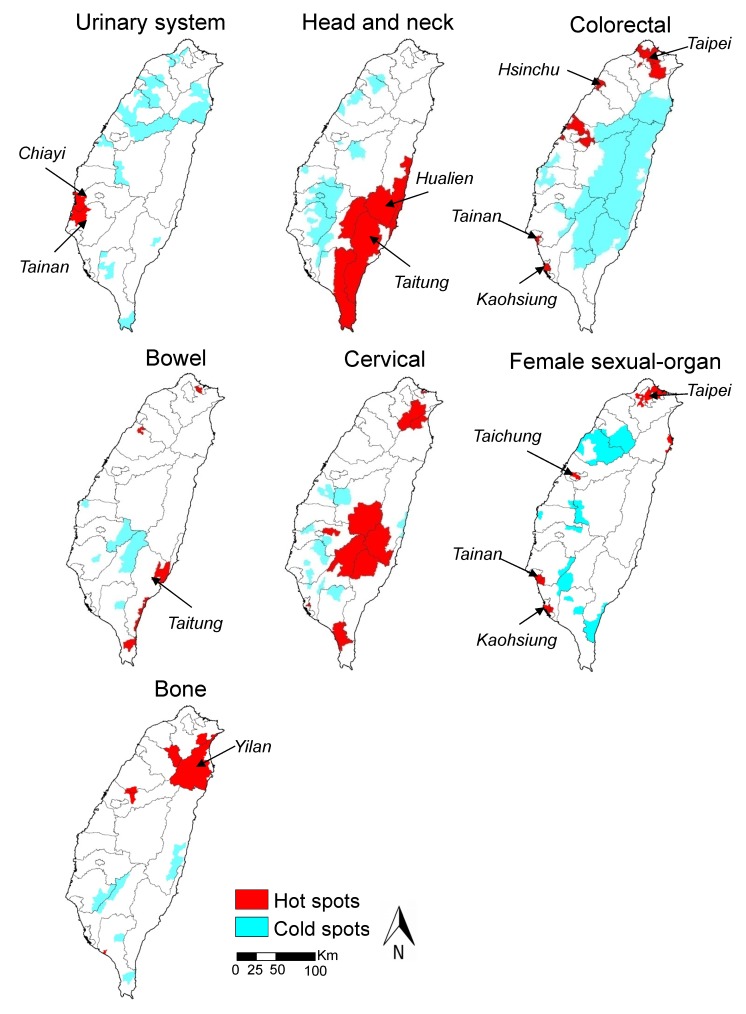
The locations of statistically significant hot spots and cold spots of major female cancer categories.

Figure 2 displays a map illustrating the geographical distribution of high and low PC scores of townships for seven major cancer categories across the Taiwan region from 1972–2001. The dark red areas on the map denote the hot spots where PC scores are relatively higher than other surrounding townships, and the light blue areas, representing the cold spots, are contrasted to those. For urinary system cancers, a single evident cluster of high PC1 scores was located on the southwest coast of Taiwan. The aggregated areas of high mortality rates of urinary system cancers in Taiwanese women were primarily scattered along the southwest coast of Taiwan in Chiayi County and Tainan County. The location of high-mortality clusters of female HNC is observable in Taitung and Hualien Counties of Eastern Taiwan, and the hot spot areas are significantly larger than the other clusters. A few scattered small clusters of high colorectal cancer mortality for females are distributed in the Northern, Central, and Southern Taiwan, including the four metropolitan areas of Taipei, Hsinchu, Tainan, and Kaohsiung. The related hot spots of bowel cancers are primarily located in the Taitung coastal regions of Southeast Taiwan. The major hot spots of female cervical cancers are focused on the inland areas of Taiwan. Small-area clusters with high-mortality rates of sexual-organ cancers in women are observable in the major metropolitan areas (Taipei, Taichung, Tainan, and Kaohsiung) across the Northern, Central and Southern Taiwan. Furthermore, a spatial aggregation of high-mortality rates exists for bone cancers among women in Yilan County, which is situated in the northeastern region of Taiwan.

### 3.4. Descriptive Statistics for the Various Variables

Table 4 is a summary table that describes the statistical difference between the geographical hot spots and cold spots in seven major cancer categories. For the five major cancer categories (Category 3–7), the average of human population densities among the hot spots were all higher than those among the cold spots, exhibiting statistically significant differences in colorectal and sexual-organ cancers, with approximately 76 and 119 people per hectare, respectively. The hot spots of female sexual-organ cancers exhibited the highest female proportion among the major seven categories, and the proportion of the female population aged 35 to 45 years was the highest at 17.9%. The highest proportion of the female population aged 50 to 70 years in the hot spots of urinary system cancers was approximately 20.3%, followed by bowel cancers with 19.6%. The hot spots of HNC had an apparently higher proportion of the aboriginal population, which was more than 20 times greater than the cold spots. Moreover, for cervical cancers, the proportion of the aboriginal population in the hot spots was nearly four times more than that in the cold spots. Regarding human behavior factors, a significantly higher average prevalence of CS and BQC was observed for the cancer hot spots, including HNC and cervical, compared with the cold spots. Among them, the BQC prevalence in the HNC hot spots was more than twice as high as in the cold spots.

Considering heavy metal concentrations in the soil between hot spots and cold spots for cancers of the urinary system, we observed that the average level of soil containing the heavy metal As in the hot spots was significantly higher than the average level in the cold spots. The average level of soil containing the heavy metal Ni in the HNC hot spots was significantly higher than the average level in the cold spots. In addition, the hot spots of colorectal and bowel cancers exhibited Cr 5 times higher than in the cold spots; however, only the difference of colorectal cancers reached a significant level. The level of the heavy metal Cr in the soil of hot spots for bone cancers was 10 times higher than in the soil of cold spots.

## 4. Conclusions

We conducted PCA and incorporated spatial autocorrelation to determine the geographical distributions and potential risk factors of the major types of cancers in Taiwanese women. We divided the 21 cancer types in women into seven major cancer categories, and separately determined the clustering patterns of geospatial hot spots with high mortality rates. Finally, we included the potentially relevant risk factors to further clarify and explain the causes of spatial distributions of hot spots for each of the major cancer categories. The first category (urinary system cancers) was associated with urinary system cancers, including bladder, kidney, skin, and lung cancers. Urothelial cell (*i.e.*, transitional cell) carcinoma is a type of cancer that typically occurs in the urinary system, involving parts of the bladder, kidney, and ureter. The risk of developing urinary system cancers increases with age and women are affected more often than are men, predominantly between the ages of 50 and 70 years [18,19].

**Table 4:** Summary of characteristic difference between the geographic hot and cold spots in seven major cancer categories.

Variables	Urinary system		Head & Neck		Colorectal		Bowel		Cervical		Female sexual organ		Bone	
	Hot (12)	Cold (44)	Hot (31)	Cold (45)	Hot (37)	Cold (37)	Hot (8)	Cold (6)	Hot (20)	Cold (26)	Hot (37)	Cold (29)	Hot (13)	Cold (12)
**Demographics**														
Pop. Density (people/ha)	7.6	13.5	0.8	7.1	75.8 ***	1.5	2.3	1.1	17.2	8.1	118.7 ***	3.2	5.6	1.0
Prop. Female (%)	48.2	47.2	45.7	47.0	49.0 ***	45.8	46.1	45.7	46.4	47.4	49.8 ***	46.0	46.6	45.8
Prop. Aborigines (%)	0.01	0.25	1.07 ***	0.05	0.14	0.89	0.76	0.51	0.51 *	0.13	0.16	0.30	0.37	0.67
Prop. F. 35~45years (%)	14.5	15.3	14.3	14.6	17.2 ***	13.8	14.1	14.1	14.9	14.7	17.9 ***	13.8	14.6	14.2
Prop. F. 50~70years (%)	20.3 **	17.4	18.3	19.6	16.6	18.5	19.6	17.7	17.0	18.9	16.9	19.3	17.6	20.5
**Human behaviors**														
BQC (per 10^5^ persons)	14.06	15.29	34.48 ***	16.04	12.26	28.52	30.08	24.33	24.52 *	18.35	11.40	18.60	13.52	23.41
CS (per 10^5^ persons)	34.83	37.51	42.88 ***	36.42	36.33	42.31	41.45	36.00	39.43	37.13	38.08	38.13	37.12	41.06
**Soil heavy metal content**														
As (mg/L)	7.7 ***	4.5	3.3	6.0	4.0	4.7	2.7	7.9	3.3	6.4	4.2	5.7	2.6	3.2
Cd (mg/L)	0.1	0.1	0.1	0.1	0.3	0.1	0.2	0.1	0.04	0.1	0.2	0.1	0.05	0.1
Cr (mg/L)	0.2	1.5	0.4	0.6	3.3 *	0.6	0.5	0.1	0.3	1.2	1.8	0.8	1.1 *	0.1
Cu (mg/L)	4.0	10.0	7.9	5.7	16.1	8.4	12.5	3.5	7.0	5.7	13.6 **	5.5	9.2	6.3
Hg (mg/L)	0.1	0.2	0.1	0.2	0.2 *	0.1	0.1	0.1	0.1	0.2	0.2	0.2	0.1	0.1
Ni (mg/L)	1.8	2.8	4.0 ***	1.9	4.1	3.9	3.5	2.1	1.7	3.3	3.2	2.7	1.2	3.1
Pb (mg/L)	6.3	10.1	7.1	7.7	10.8	8.9	10.8	5.0	9.6 *	6.8	10.6 *	7.9	12.8 **	7.4
Zn (mg/L)	8.8	15.2	9.9	10.0	20.8	10.3	10.9	6.1	8.8	11.0	16.6	10.0	10.1	8.3

Notes: (1) Urinary system cancers include bladder, kidney, skin(non-melanoma), and lung cancers; head & neck cancers include esophagus, larynx, nasopharynx, and oral cavity cancers; colorectal cancers include malignant tumors of the colon and/or the rectum; bowel cancers include rectum and intestine cancers; female sexual organs cancers include breast and ovarian cancers. (2) The number of townships is shown in parentheses. (3) Abbreviations: BQC, betel quid chewing; CS, cigarette smoking; Hot, hot spots; Cold, cold spots. (4) The average value of a given variable in hot spots is significantly larger than that in cold spots, and the significance is determined by a Student’s *t*-test: * *p* < 0.05, ** *p* < 0.01, *** *p* < 0.001.

The evidence strongly supports a causal relationship between environmental exposure to As levels and the occurrence of specific cancers including bladder, kidney, lung, liver, and skin [20,21,22]. A dose-response relationship exists between As ingestion from drinking water and human cancers of the bladder, lung, and kidney in the black foot disease (BFD) endemic area of Southwest Taiwan [23,24]. The natural source of As in soil is parent material in Taiwan. Arsenic can be released to the environment through the natural weathering and breakdown of rocks. Arsenic contamination of agricultural soils due to long-term irrigation with contaminated groundwater, and showed that this lead to phyto-accumulation in food crops, and hence risk to human from dietary exposure to As. In summary, the consistent findings from the first category of this study demonstrate that the hot spots of urinary system cancers occur where people are most likely to be exposed to environmental media (e.g., groundwater, soil) with high levels of As.

The second category of HNC was composed of esophagus, larynx, nasopharynx, and oral cavity cancers. The HNC hot spots, primarily concentrated in Hualien and Taitung Counties of Eastern Taiwan, exhibited the largest proportion of the aboriginal population and the highest prevalence rates of CS and BQC. During the 2007–2013 period, Hualien County was home to 17.9% of the entire aboriginal population, accounting for the largest proportion of the aboriginal population in Taiwan, followed by Taitung County, with 15.7% [25]. However, Taiwan aborigines have a traditional ethnic habit of betel quid use, and thus a higher prevalence of BQC. The International Agency for Research on Cancer (IARC) identified the intake of BQC with tobacco and tobacco smoking as human carcinogens that act on target organs, including the oral cavity, pharynx, larynx, and esophagus [26]. Numerous studies have confirmed both CS and BQC as culprits for HNC [27,28,29,30,31]. The result of this study showed that higher levels of Ni in the soil of HNC hot spots, is consistent with the close relation of Ni with oral cancer from previous studies [1,10,32].

Urbanization refers to concentrated human populations, and the degree of urbanization can be directly measured based on population density. A link between urbanization and health has recently attracted increased attention and has been reported for diverse populations and regions. Urbanization results in changes of dietary practices, lifestyle, and occupation, which increases the release of anthropogenic chemicals into the environment. Areas of high-level urbanization increase the prevalence of lifestyle diseases such as cancer. The hot spots of colorectal and female sexual-organ cancers were located in areas of high levels of urbanization or high population density. Urbanization might be associated with increased risk of colorectal cancer [33,34], which is growing rapidly in China because of the influence of urbanization [35]. Among the cancers of the female sex organs, the incidence rates of breast cancer are higher in urban areas compared with rural areas in the United States [36]. Rapid urbanization and the adoption of a western diet and lifestyles are culprits for increasing the risk of breast cancer in developing countries [37,38]. The hot spots of colorectal cancer also exhibited high levels of Cr in the soil, which is consistent with the proposed role for Cr (VI) in promoting colorectal cancer [39].

For the Singapore Chinese, CS is associated with an increased risk of rectal cancer [40], and the results derived from our study indicated that the hot spots of bowel cancers, including rectal cancer, exhibited a high average prevalence of CS. The proportion of the Taiwanese aboriginal population in the hot spots of cervical cancer was second only to the HNC hot spots; therefore, they had a high prevalence of BQC and CS. However, smoking has been determined to be a risk factor for cervical cancer among human papillomavirus (HPV) positive women [41]. Aboriginal women exhibited 3.6 times greater incidence rates of invasive cervical cancer than non-aboriginal women did, and one of the main reason is that they do not participate in routine cervical-cancer screening as frequently as do non-aboriginal women [42]. The levels of soil Cr in the hot spots of bone cancers were apparently higher than in the cold spots. Animal and human studies have reported that Cr (VI) can cause an increased risk of bone cancer [43].

In conclusion, based on the overall consistency of our study results and those of previous studies regarding high-risk locations and related risk factors for major types of female cancers in Taiwan, cancer has clearly become a major threat to public health in Taiwan. The comprehensive cancer control programs should be implemented and evaluated in these hot spot areas. However, in order to accomplish the goal of early detection and treatment, the Taiwan’s government has taken many actions. From 2010, the Health Promotion Administration, Ministry of Health and Welfare, R.O.C. (Taiwan) is massively promoting four cancer-screening programs in all hospitals, for breast cancer, colon cancer, cervical cancer, and oral cancer. Furthermore, considerable attention should be directed toward areas with high mortality rates of specific cancer types, and the government should allocate additional financial resources to health care to prevent unnecessary costs. The oral cancer-screening program should be especially advocated in the HNC hot spots of Hualien County and Taitung County. The colon and breast cancer-screening programs should be put into practice as soon as possible in the major metropolitan areas, such as Taipei City, Taichung City, and Kaohsiung City. In addition, more efforts are needed on the implement of cervical cancer-screening program for inhabitants in the Taiwan mountainous areas. Intensive research on cancer should also be conducted for planning effective cancer control programs.
